# An Adaptive Compensation Algorithm for Temperature Drift of Micro-Electro-Mechanical Systems Gyroscopes Using a Strong Tracking Kalman Filter

**DOI:** 10.3390/s150511222

**Published:** 2015-05-13

**Authors:** Yibo Feng, Xisheng Li, Xiaojuan Zhang

**Affiliations:** School of Automation and Electrical Engineering, University of Science and Technology Beijing, Beijing 100083, China; E-Mails: feng_yibo@163.com (Y.F.); zxjjianghan@163.com (X.Z.)

**Keywords:** strong tracking Kalman filter, bias, compass, MEMS gyroscope

## Abstract

We present an adaptive algorithm for a system integrated with micro-electro-mechanical systems (MEMS) gyroscopes and a compass to eliminate the influence from the environment, compensate the temperature drift precisely, and improve the accuracy of the MEMS gyroscope. We use a simplified drift model and changing but appropriate model parameters to implement this algorithm. The model of MEMS gyroscope temperature drift is constructed mostly on the basis of the temperature sensitivity of the gyroscope. As the state variables of a strong tracking Kalman filter (STKF), the parameters of the temperature drift model can be calculated to adapt to the environment under the support of the compass. These parameters change intelligently with the environment to maintain the precision of the MEMS gyroscope in the changing temperature. The heading error is less than 0.6° in the static temperature experiment, and also is kept in the range from 5° to −2° in the dynamic outdoor experiment. This demonstrates that the proposed algorithm exhibits strong adaptability to a changing temperature, and performs significantly better than KF and MLR to compensate the temperature drift of a gyroscope and eliminate the influence of temperature variation.

## 1. Introduction

MEMS inertial sensors are more widely used in the middle- and low-level market because of their advantages such as diminutive size, low cost, and low power consumption [1]. However, the temperature variation reduces the precision of the MEMS gyroscope seriously when the MEMS gyroscope is used in a changing environment such as in the car or unmanned aerial vehicle without a constant temperature adjustment [2], and this feature makes it necessary to model and compensate for the bias drift (called “drift” for short) of the MEMS gyroscope to increase the accuracy.

The noise in an MEMS gyroscope is composed of two components: a slow-changing component and a high-frequency component with an average of zero. The gyroscope is sensitive to temperature variations, so the surrounding temperature variation leads to the bias drift of the gyroscope. Then, as the error of the angular velocity, the drift causes error accumulation in the orientation, thus, this is the major part of the slow-changing component [3]. The drift is not linear with temperature, therefore, the model is not entirely accurate. Additionally, different MEMS gyroscopes may have the same drift model, however, because of the differences in the respective production process, the parameters of each gyroscope vary between one another. Thus, the result is not accurate if we use the same parameters to compensate the drifts of all gyroscopes. However, the cost and time of calibration will increase if we calibrate the parameters of every MEMS gyroscope, and the parameters will be also changed to affect the accuracy of the gyroscope when the supply voltage or work environment changes [4]. Thus, we present an adaptive algorithm for a system integrated with MEMS gyroscopes and a compass to compensate the drift of MEMS gyroscopes for different temperatures and work environments and to improve the accuracy of the MEMS gyroscope.

Before explaining our algorithm, it is necessary to briefly discuss several other approaches for compensating the drift of a MEMS gyroscope. Kirkko-Jaakkola *et al*. demonstrated that temperature variation greatly affects the bias of the MEMS gyroscope [5]. Consequently, temperature drift compensation should be implemented to reduce the influence of temperature. There are two common ways to implement the compensation. The first way is using a temperature control system to keep the temperature stable. Because of the stable temperature, the bias is not influenced by temperature, so this method has high precision and reduces the computational burden, and also makes the use of the temperature-sensitive instruments much easier, but the cost, power consumption and size are all increased [6]. The second way is using signal processing to compensate the temperature drift. This way does not increase the size, weight and power consumption, and it analyses the characteristics of the drifts, then uses a suitable model to compensate the drift, but the precision depends on the accuracy of the model and parameters. As we know, the relation between the drift and the temperature is nonlinear, so if the model and parameters are more accurate, the compensation precision is higher, but the more complex model increases the computational burden.

Temperature drift modeling and compensation is used much more widely than the first way. Firstly, the model of the temperature drift is built by multiple linear regression (MLR) on the basis of the structure of the gyroscope and experimental data. Then, calibration experiments are necessary to obtain the available data. Finally, algorithms such as the least squares method, or polynomial segmentation fitting are used to calculate the parameters of the model. Afterwards, temperature compensation can be implemented [7,8,9,10,11]. These methods are easy to implement and compensate the drift in the full temperature range. However, the main drawback of these approaches is that the relationship between the bias of the gyroscope and the temperature is complicated and nonlinear, so the model is not completely accurate, so the fixed parameters cannot maintain high compensation precision over the full temperature range. Additionally, if the working environment is altered, the accuracy will be affected, because the parameters do not change to adapt to the environment. To reduce the error caused by inaccuracy of the model and improve the fault-tolerance, the artificial neural network is used for the temperature compensation of gyroscope bias, but the features of the neural network, such as low convergence speed and easily falling into local minima, limit the compensation accuracy [12,13]. Some methods, such as Kalman Filter (KF), extended Kalman Filter (EKF), iterated unscented Kalman Filter (IUKF), can be used for neural network training to reduce the influence of these features and improve the compensation precision [14,15]. KF, EKF, UKF are the most common methods for integrated navigation systems to compensate various errors which include gyroscope drift. The compensation precision is high and the estimations of errors change after each iteration to match the environment when each navigation subsystem works properly, so the gyroscope drift is compensated accurately with the support of the other navigation subsystems [16,17,18], but the estimation of gyroscope drift changes only after iteration. Once a fault occurs at some subsystem, the iteration may stop to wait for the fault removal, and the drift estimation also remain unchanged, so faults have a great influence on the compensation precision.

In order to solve the problem that the model is not completely accurate, decrease the influence of faults and accurately compensate the MEMS gyroscope drift, we build a simple drift model and estimate suitable parameters for the current environment after each iteration, so an adaptive compensation algorithm based on STKF is presented in this paper. Firstly, a simplified bias model is built to reduce the amount of calculation. Then the STKF is built on the basis of the simplified bias model, and the bias model parameters are set as state variables of STKF, so these parameters are estimated accurately under the support of a compass, and these parameters make the simplified model maintain high precision in the current environment. When the STKF is stopped for some reason, such as a fault or magnetic interference, the bias drift is also compensated accurately by the model and parameters. This algorithm is different from the other algorithms in that it can estimate the most appropriate parameters for the simplified model in the current environment. If the environment does not change too much, these parameters can maintain a high precision of bias estimation. The parameters change adaptively with the environment, thus, they have a strong adaptability to the changing environment, which makes the compensated gyroscope unaffected by the environment.

The system configuration is introduced in Section 2. Section 3 provides a detailed description of the adaptive compensation algorithm. Section 4 presents the experimental results of this algorithm, and finally, the conclusion of this paper is presented in Section 5.

## 2. System Configuration

The system is integrated with a triaxial compass, three MEMS gyroscopes (ADXRS623), and a triaxial accelerometer. The triaxial compass is composed of a single-axis magnetoresistive sensor (HMC1021Z) and a dual-axis magnetoresistive sensor (HMC1022), which are used to measure the axial intensity of the magnetic field for every axis. The triaxial accelerometer is composed of a single-axis accelerometer (ADXL103) and a dual-axis accelerometer (ADXL203), which are used to measure the axial intensity of the acceleration for every axis to calculate the attitude angle of the system. Then, the orientation of the compass can be calculated from the axial strength of the magnetic field, which is compensated by the attitude angle of the system [19]. The characteristics of these sensors are shown in Table 1. The orientation is calculated using the arctangent of the axial intensity of the magnetic field which is compensated by the axial intensity of the magnetic field on z-axis and attitude angles, so the error is not accumulated. Additionally, the impacts of temperature variation on the magnetoresistive sensors are at almost the same proportion, so the arctangent can mostly eliminate the impact from temperature variation. The orientation of the compass can be used to assist in the compensation of the MEMS gyroscope bias drift.

**Table 1:** The characteristics of sensors.

	ADXRS623	HMC1022/1021	ADXL103/203
Measurement range	±150°/s	±6 gauss	±1.7 g
Sensitivity	12.5 mv/°/s	1 mv/v/gauss	1000 mv/g
Sensitivity change due to temperature	±3%	−0.3%/°C	±0.3%
Noise Density	0.04°/s/√hz	48 nv/√hz	110 μg/√hz rms

## 3. The Adaptive Compensation Algorithm

In this paper, we lump all slow-changing errors together, regardless of whether they are caused by physical phenomena or temperature sensitivity, and call them collectively ‘drift’. The system is always used in many types of environments, and the working temperatures are also different, thus, a temperature compensation model is quite necessary, and the errors caused by other error sources can be treated as constants in a short period of time. Therefore, a model of the MEMS gyroscope bias drift can be constructed as the model below, and an STKF is used to estimate the parameters of model.

### 3.1. The Model of MEMS Gyroscope Drift

The model of the uncompensated angular velocity of the MEMS gyroscope is:

(1) $$\omega =\omega_{t}+B_{d}+n$$

where:

ω—measurement of angular velocity.
$\omega_{t}$—true but unknown angular velocity.
$B_{d}$—slow-changing component of the signal; this is the gyroscope drift.
n—stochastic component of the signal.

In this paper, the precision of the gyroscope is impacted by $B_{d}$ and n. We know that $B_{d}$, as the slow-changing component, is mainly caused by physical phenomena and temperature variation, so a temperature-compensating model is needed, and errors caused by the other error sources can be treated as constant in a short period of time. The model of $B_{d}$ can be constructed [20].

The slow-changing component of the gyroscope is not only related to the measured temperature of the MEMS gyroscope but is also related to the temperature gradient of the surroundings. Additionally, the temperature gradient and the rate of temperature variation have a linear relationship; therefore, the slow-changing component of the MEMS gyroscope can be modeled as:

(2) $$B_{d}=a*T+b*T\prime +c$$

where:

T—measured temperature of the gyroscope.
T′—rate of temperature variation.
a, b, c—parameters of the model [21].

### 3.2. The STKF for Model Parameters

The accurate estimated value of the slow-changing component of the MEMS gyroscope requires accurate values of a, b, and c in real time. Consequently, the STKF is modeled on the basis of Equation (2), and a, b, and c are all state variables of the STKF [22]. The STKF is as follows:

(3) $$\begin{array}{l} x_{k+1}={\text{Φ}}_{\left(k+1,k\right)}*x_{k}+{\text{Γ}}_{\left(k+1,k\right)}*\omega_{k}\\ y_{k+1}=H_{k+1}*x_{k+1}+v_{k+1} \end{array}$$

where:

$x_{k+1}$—state variables of the STKF at time *k* + 1 and also the parameters of the slow-changing component at time *k* + 1, $x_{k+1}={\left[\begin{array}{c} a\\ b\\ c \end{array}\right]}_{k+1}$.
$y_{k+1}$—measurement of the STKF at time *k* + 1, $y_{k+1}=\left[\begin{array}{c} m_{k+1}\\ m_{k}\\ m_{k-1} \end{array}\right]$. As the initial values of $y_{k+1}$, $y_{1}$, $y_{2}$ and $y_{3}$ is measured when the gyroscope is motionless in the beginning.
$m_{k+1}$—measurement of the gyroscope drift at time *k* + 1.
  (4) $$m_{k+1}={\hat{z}}_{k}+\text{Δ}A_{\left(k+1,k\right)}/\text{Δ}t$$
${\hat{z}}_{k}$—estimated value of the gyroscope drift based on the parameters at time *k*.
  (5) $${\hat{z}}_{k}=a_{k}*T_{k}+b_{k}*T\prime_{k}+c_{k}$$
$\text{Δ}A_{\left(k+1,k\right)}$– variation of the difference between the orientation of the compass and the orientation of the MEMS gyroscope from time k to time *k* + 1.

(6) $$\text{Δ}A_{\left(k+1,k\right)}=(A\_compass_{\left(k+1\right)}-A\_Gyro_{\left(k+1\right)})-(A\_compass_{\left(k\right)}-A\_Gyro_{\left(k\right)})$$

where: $A\_compass_{\left(k+1\right)}$, $A\_Gyro_{\left(k+1\right)}$, $A\_compass_{\left(k\right)}$, $A\_Gyro_{\left(k\right)}$ are the orientations of compass and MEMS gyroscope at time *k* + 1 and time *k* respectively.

Δt—time interval from time k to time *k* + 1.
$H_{k+1}$—measurement matrix of the STKF, which can be calculated by the measured temperature and the rate of temperature variation. $H_{k+1}=\left[\begin{array}{c} \begin{array}{ccc} T_{k+1} & \;T\prime_{k+1}\; & 1 \end{array}\\ \begin{array}{ccc} \;T_{k}\; & \;T\prime_{k}\; & 1 \end{array}\\ \begin{array}{ccc} T_{k-1} & \;T\prime_{k-1}\; & 1 \end{array} \end{array}\right]$. As the initial values of $H_{k+1}$, $H_{1}$, $H_{2}$ and $H_{3}$ is measured when the gyroscope is motionless in the beginning.
$T_{k+1}$—measured temperature of the gyroscope at time *k* + 1.
$T\prime_{k+1}$—rate of temperature variation at time *k* + 1; for ease of use, it is simplified as: $T\prime_{k+1}=(T_{k+1}-T_{k})/\text{Δ}t$.

The parameters of bias drift model change with the environment, so if the environment doesn’t change too much, the parameters also don’t change sharply. Then we can make an assumption that the parameters are unchanged in short time, so the matrices ${\text{Φ}}_{\left(k+1,k\right)}$ and ${\text{Γ}}_{\left(k+1,k\right)}$ are all identity matrix.

Because of the inaccuracy of the model and noise characteristics, the correction effect of the new measurements on the estimated value decreases with time, the correction effect of the early measurements increases, and the error of the estimated value of the traditional KF increases. Thus, the STKF is used to increase the weight of the new measurements to avoid the error of the estimated value increase with time.

Increasing the variance of the measurement noise *R* and the initial value of the covariance matrix P_0_ can decrease the influence from early measurements on the latest state variable; thus, the noise characteristics are necessary to be changed.

The characteristics of the measurement noise are modified as:

(7) $$\begin{array}{c} E[v_{k}^{N}]=0\\ Cov[v_{k}^{N}{\left(v_{k}^{N}\right)}^{T}]=R_{k}^{N}=R_{k}*s^{N-k}(s>1) \end{array}\}$$

where:

$R_{k}^{N}$—modified variance of the measurement noise at time *k*, which is used to calculate the state variable x*^N^* at time *N*.
$R_{k}$—original variance of measurement noise at time *k*.

${\hat{x}}^{N}$—the estimation of state variables at time *N*, which recursively obtained from ${\hat{x}}^{1}$ to ${\hat{x}}^{N-1}$, so the measured values y^1^ to y*^N^* are all useful to calculate the state variable ${\hat{x}}^{N}$; therefore, ${\hat{x}}^{N}$ is a linear combination of the measured values, and $R_{1}^{N}$ to $R_{N}^{N}$ are all useful in calculating ${\hat{x}}^{N}$. If the characteristics of the measurement noise in Equation (7) are used, the longer the time interval span from time *k* to time *N*, the larger the value of $R_{k}^{N}$ is, and the relevant gain matrix $K_{k}$ becomes smaller. This means that the earlier time implies that the weight of measurement used for calculating ${\hat{x}}^{N}$ is smaller; therefore, y*^k^* contributes less to ${\hat{x}}^{N}$.

The initial state characteristic is modified as:

(8) $$\begin{array}{c} E[x_{0}^{N}]=m_{x_{0}}\\ Var[x_{0}^{N}]=P_{0}^{N}=P_{0}*s^{N}(s>1) \end{array}\}$$

The processing noise characteristic is modified as:

(9) $$\begin{array}{c} E[\omega_{k-1}^{N}]=0\\ Cov[\omega_{k-1}^{N}{\left(\omega_{k-1}^{N}\right)}^{T}]=Q_{k-1}^{N}=Q_{k-1}*s^{N-k}(s>1) \end{array}\}$$

The noise characteristic of the traditional KF is replaced by Equations (7)–(9) to obtain the STKF:

(10) $$\begin{array}{c} {\hat{x}}_{k/k-1}^{*}={\text{Φ}}_{\left(k,k-1\right)}{\hat{x}}_{k-1}^{*}\\ {\hat{x}}_{k}^{*}={\hat{x}}_{k/k-1}^{*}+K_{k}^{*}(y_{k}-H_{k}{\hat{x}}_{k/k-1}^{*})\\ K_{k}^{*}=P_{k/k-1}^{*}H_{k}^{T}{\left(H_{k}P_{k/k-1}^{*}H_{k}^{T}+R_{k}\right)}^{-1}\\ P_{k/k-1}^{*}={\text{Φ}}_{\left(k,k-1\right)}(sP_{k-1}^{*}){\text{Φ}}_{\left(k,k-1\right)}^{T}+Q_{k-1}\\ P_{k}^{*}=(I-K_{k}^{*}H_{k})P_{k/k-1}^{*} \end{array}\}$$

Equation (10) shows that the difference between the STKF and the traditional KF is only the attenuation factor s. When *s* > 1, $P_{k/k-1}$ is greater than that of the traditional KF, and $K_{k}$ becomes larger as well. Thus, ${\hat{x}}_{k}=(1-K_{k}H_{k}){\hat{x}}_{k/k-1}+K_{k}y_{k}$ shows that the influence from the old measurements on the estimated value is decreased, and the weight of the new measurement increases [23,24].

The above approach estimates the slow-changing component of the MEMS gyroscope accurately, and the parameters are unaltered in a short period of time; therefore the STKF is implemented at a certain frequency. A higher frequency will yield better results for the STKF, however, the noise of the compass causes errors in $\text{Δ}A_{\left(k+1,k\right)}$ and also causes the measured value of the STKF to contain the error. If the frequency decreases, the time interval Δt of the STKF will increase, and the impact of the compass noise will be reduced as a result of $y_{k+1}={\hat{z}}_{k}+\text{Δ}A_{\left(k+1,k\right)}/\text{Δ}t$. Considering the two abovementioned aspects, the interval for STKF implementation is set every 10 s. Because the environment does not change significantly in 10 s, the parameters are not altered significantly in that time, which can guarantee accuracy. Also the long time interval reduces the amount of calculation.

## 4. Experimental Results

This section presents the results of the two temperature experiments. For ease of implementation, the first experiment was operated in the laboratory with a large change of temperature and in the second experiment, the system was installed on a vehicle to test its performance when the vehicle moved in an outdoor environment.

### 4.1. The Static Experiment

In the first experiment, the system is motionless during the entire process, and the temperature is increased from 23 °C to 58 °C at a non-uniform speed and then lowered to test the performance of this algorithm. The magnetic interference can be identified via the insensitivity of the MEMS gyroscope to magnetic interference; thus, the STKF is only carried out under the condition in which there is no magnetic interference.

**Figure 1:**
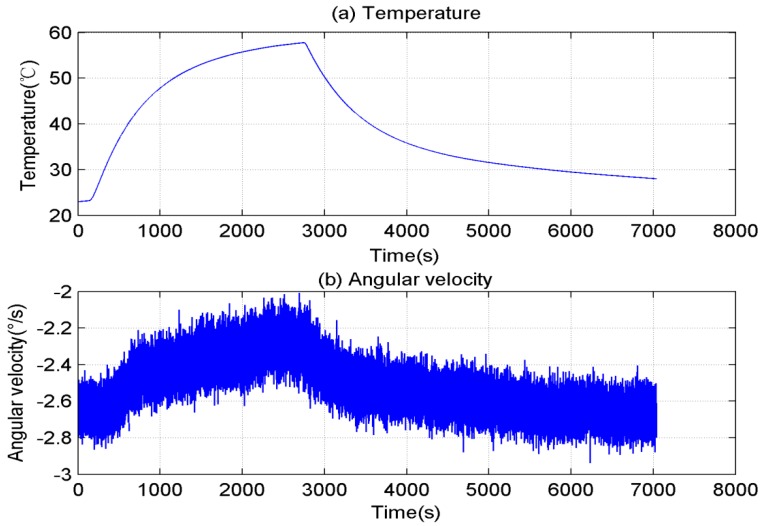
Original data of motionless gyroscope. (**a**) Temperature variation; (**b**) Angular velocity.

Figure 1 shows that when the gyroscope temperature increased from 23 °C to 58 °C and then lowered to ambient temperature, the motionless gyroscope bias drifted with temperature. The gyroscope signal changed rapidly when the temperature varied quickly. Thus, the error of the gyroscope accumulated rapidly if the bias was not compensated accurately.

**Figure 2:**
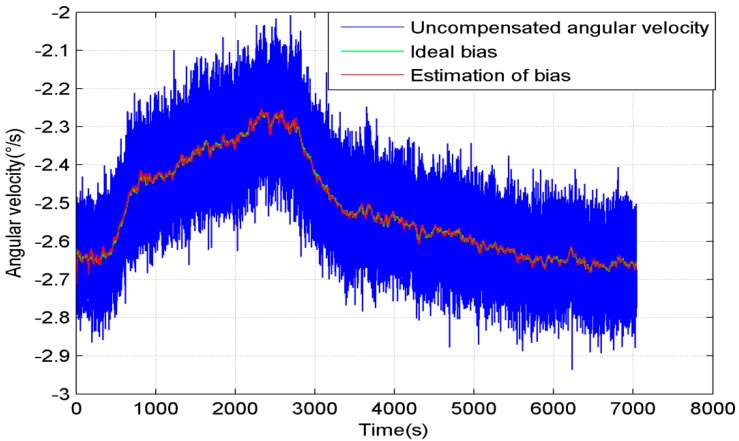
Uncompensated angular velocity and the estimation of the bias of the gyroscope.

The blue line in Figure 2 is the uncompensated angular velocity of the motionless gyroscope, the red line is the estimation of the gyroscope bias, and the green line is the ideal bias. We can see that the bias estimation follows the ideal bias very well.

**Figure 3:**
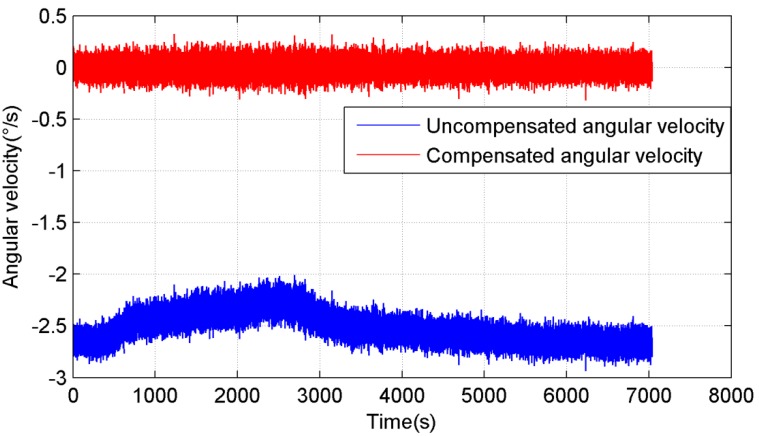
Uncompensated and compensated angular velocity of the gyroscope.

In Figure 3, the blue line is the uncompensated angular velocity of the gyroscope, and the red line is the compensated the angular velocity. It can be seen that the compensated angular velocity of the gyroscope is not affected by the temperature.

**Figure 4:**
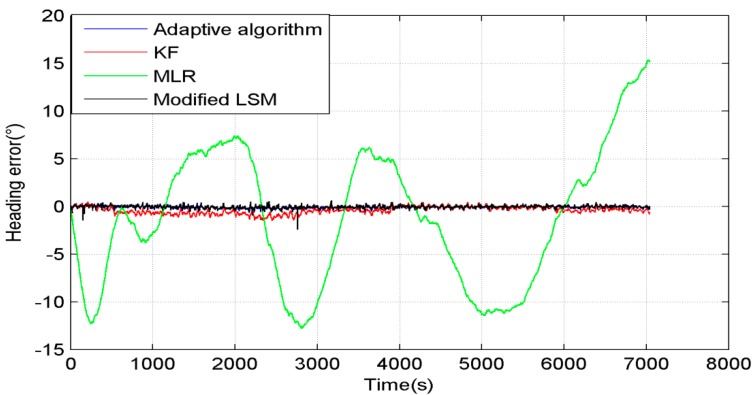
Heading error of different algorithms without interference.

Figure 4 shows comparison of the heading error by using different algorithms in the entire process. The blue line is the heading error compensated by the adaptive algorithm, and the error is kept in the range from −0.6° to 0.4°. The red line shows the heading error compensated by Kalman Filter (KF), and the error is kept in the range from −1.5° to 0.5°. The green line is the heading error compensated by MLR. The parameters are calculated by the least squares method (called “LSM” for short) using data in the entire process. The heading error is much larger than the other methods, and the error is kept in the range from −12.5° to 15°. The black line is the heading error compensated by the modified LSM. It calculates the parameters every 10 s to make these three parameters change with environment and it almost overlaps with the blue line.

**Figure 5:**
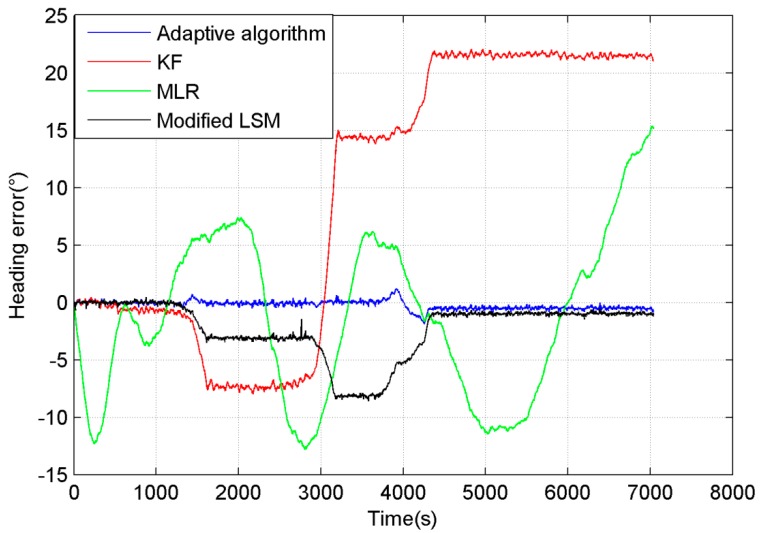
Heading error of different algorithms with interference.

The adaptive algorithm does not work all the time, for example magnetic interference in the surroundings may cause some errors in the system, so this algorithm does not run to estimate the parameters. To test the temperature compensation performance of different algorithms when the algorithms do not work for some reason, we make the algorithms not work in three time periods (300 s, 300 s and 600 s). The results are shown in Figure 5. The blue line is the heading error which is compensated by the adaptive algorithm, where it can be seen that the algorithm also compensates the drift accurately when it is in the outage, and the maximum error is just less than 1.8°. The red line is the heading error which is compensated by KF, where the heading error increases significantly during the KF outage. The green line is the heading error which is compensated by MLR. The black line is the heading error which is compensated by the modified LSM. It shows that the adaptive compensation algorithm has a much higher precision than these three algorithms, when the algorithms are in outages for some reason.

**Figure 6:**
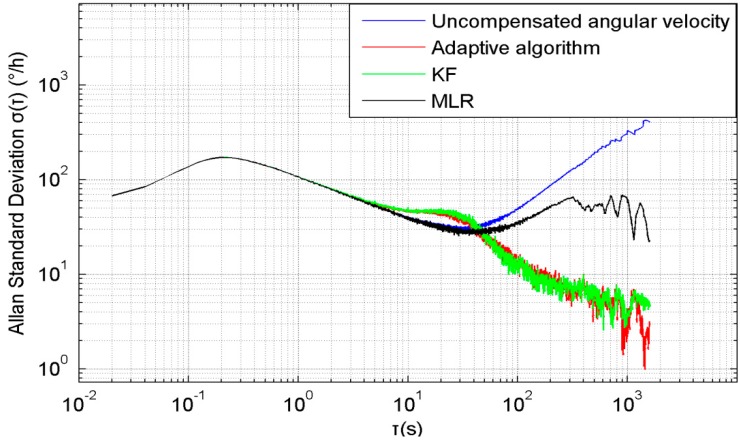
Allan standard deviation of uncompensated and compensated angular velocity error.

On the basis of the experimental data, the Allan variance is used to analyse the performance of the algorithms, as shown in Figure 6. The blue line in Figure 6 indicates the Allan standard deviation of the uncompensated angular velocity error, and the red line indicates the Allan standard deviation of the angular velocity error which is compensated by the adaptive algorithm. The green line indicates the Allan standard deviation of the angular velocity error which is compensated by KF. The black line indicates the Allan standard deviation of the angular velocity error which is compensated by MLR. It can be seen that the rate random walk (RRW) and rate ramp (RR) are obviously decreased [25,26]. The comparison of these lines shows the adaptive algorithm and KF compensate the temperature drift significantly better than the MLR. If there is no magnetic interference, the KF and adaptive algorithm have a similar performance. This adaptive algorithm significantly decreases the influence from the temperature and makes the MEMS gyroscope much more accurate and stable. Figure 6 also shows that the noise, which was at approximately τ = 10 s, is not significantly decreased, which is because the STKF is implemented every 10 s, this makes the parameters change every 10 s, and the errors caused by the noise of compass and gyroscope are also different every 10 s, therefore, this still needs further improvement.

### 4.2. The Dynamic Experiment

**Figure 7:**
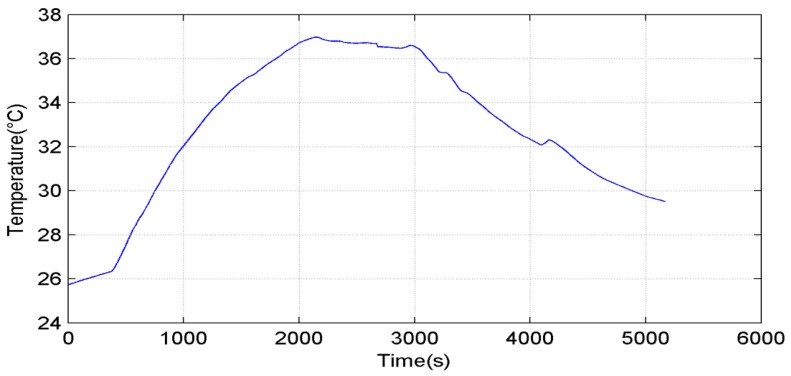
Temperature variation in the entire process.

After the static experiment, we installed the system on a vehicle, and moved outdoors to test the dynamic performance. To reduce the influence from pitching and rolling angles, the vehicle ran on a flat road. A fiber-optic gyroscope (FOG) (XW-GS1800) was fixed together with the system as the benchmark for the orientation. Figure 7 shows the temperature of the MEMS gyroscope in the dynamic experiment. The gyroscope temperature was influenced by the surrounding temperature, and changed from 26 °C to 37 °C.

**Figure 8:**
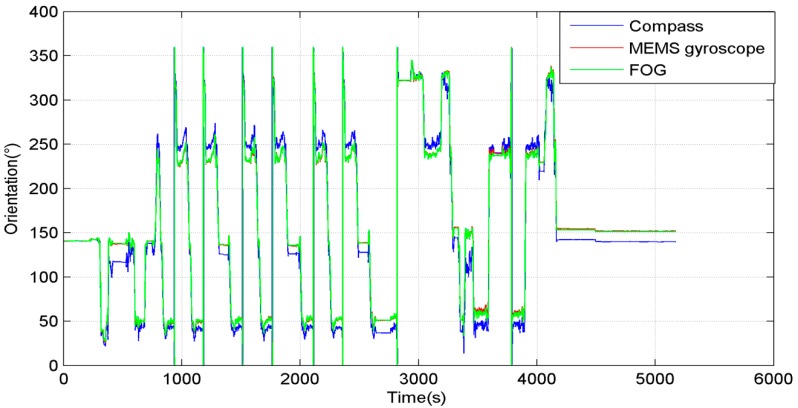
Orientation in the entire process.

The orientation in the entire process is shown in Figure 8. During most of the time, the vehicle moved around a rectangular area. The blue line is the compass orientation, the red line is the MEMS gyroscope orientation, and the green line is the FOG orientation. It can be seen that the MEMS gyroscope orientation followed FOG very well. The relative rotation angle of compass was not accurate, but when the system moved straight, and the heading error of the compass remained stable, so the adaptive algorithm only worked when the system moved straight.

**Figure 9:**
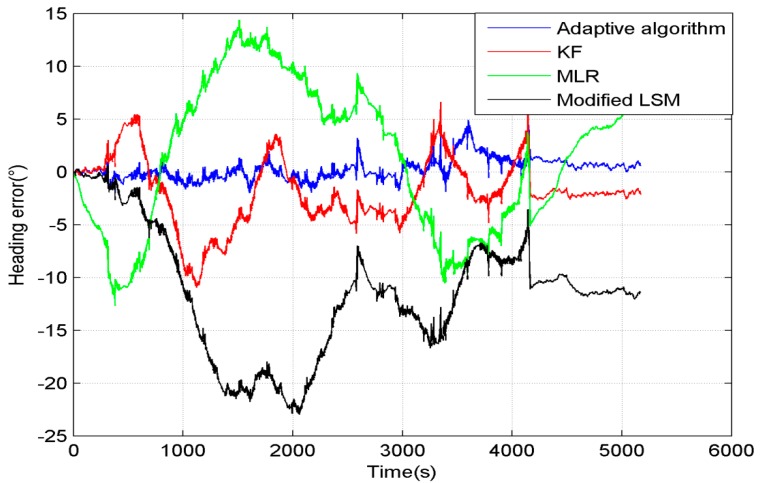
Heading error of different algorithms in the entire process.

The comparison of the results compensated by different algorithms is shown in Figure 9. The result of MLR is shown as a green line in Figure 9, and the heading error remains in the range from 15° to −13°. The KF improved the compensation precision to the range from 5° to −10°, and it is shown as a red line in Figure 9. The modified LSM didn’t compensate the bias very well, because the measurement error had a great influence on its precision, as shown as a black line in Figure 9. The compensation precision of the adaptive algorithm is better than the former algorithms. Its heading error is shown as the blue line, and it is kept in the range from 5° to −2°.

These two experiments results demonstrate that this adaptive algorithm can compensate the temperature drift much more accurately than the KF, MLR and modified LSM. The adaptive algorithm estimates the parameters of the drift model. If the algorithm did not work for some reason such as magnetic interference, the parameters which were appropriate for the current environment could also maintain the high accuracy for a short time.

In Table 2, we compare the compensation results of the four methods in the experiment. In the static experiment, the compensation accuracy of MLR is not affected by interference. As shown in the table, the precision is improved to 0.002134°/s with the compensation by MLR. When there is no interference in the static experiment, the KF improved the compensation precision to −1.1496 × 10^−4^ °/s, while the modified LSM improved it to −2.4515 × 10^−4^ °/s, and the adaptive algorithm improved this precision to 2.1619 × 10^−5^ °/s. When interference existed in the static experiment, the adaptive algorithm also performed better than the other three methods, and the compensation precision is 1.7511 × 10^−4^ °/s. In the dynamic experiment, we used the FOG as the orientation benchmark to test the performance of these four methods. The mean of the heading error which was compensated by MLR is 1.4882°. The performance of KF and modified LSM were not as good as MLR, and they are −1.9085° and −11.5318°, respectively. The adaptive algorithm improved this precision to 0.2743°, and the heading error compensated by the adaptive algorithm was much more stable than with the other methods.

**Table 2:** Comparison of compensation results of the four methods.

		MLR	KF	Modified LSM	Adaptive Algorithm
Bias drift error in static experiment without interference	Mean (°/s)	0.002134	−1.1496 × 10^−4^	−2.4515 × 10^−4^	2.1619 × 10^−5^
	Variance	5.5005 × 10^−4^	7.5105 × 10^−5^	1.1416 × 10^−4^	6.7660 × 10^−5^
Bias drift error in static experiment with interference	Mean (°/s)	0.002134	0.003023	−2.0156 × 10^−4^	1.7511 × 10^−4^
	Variance	5.5005 × 10^−4^	3.4361 × 10^−4^	1.8643 × 10^−4^	9.3155 × 10^−5^
Heading error in dynamic experiment	Mean (°)	1.4882	−1.9085	−11.5318	0.2743
	Variance	46.9166	9.8503	34.7090	0.9926

### 4.3. The Simulation of SF

The scale factor (called “SF” for short) is as important as bias drift in actual use, so it must be compensated accurately. There still are some unsolved problems for us to adaptively compensate both SF and bias drift in actual use, so we just use the data which is collected under the conditions with stable temperature and magnetic field to reduce the influence from magnetic interference. This data is combined with the data of bias and temperature in the static experiment. Then we generated the data of SF base on the information of this gyroscope datasheet. The compensation methods for bias and SF are all the adaptive algorithm. We used the static data to compensate the bias drift, and used the rotational data to compensate SF. The temperature in the simulation is same as in the static experiment.

**Figure 10:**
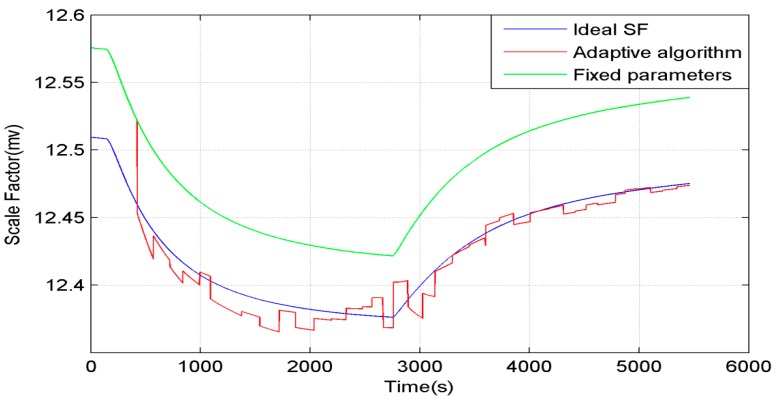
The SF in simulation.

The SF is shown in Figure 10. The blue line is the ideal SF, The green line is the estimation of SF which is calculated with fixed parameters. The red line is the adaptive estimation of SF. It can be seen that the adaptive estimation of SF follows the ideal SF very well.

**Figure 11:**
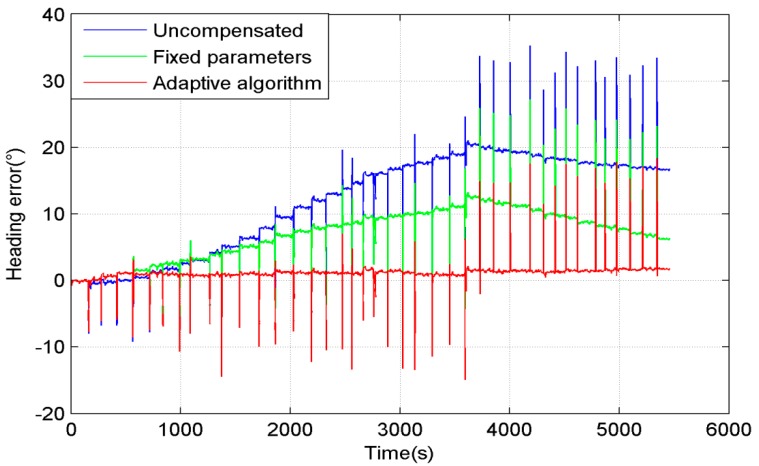
The heading error in simulation of SF.

The heading error in the entire process is shown in Figure 11. The blue line is the heading error without SF compensation. The green line is the heading error which is compensated by SF with fixed parameters. The red line is the heading error which is compensated by the adaptive estimation of SF. The reason of the heading error spikes during rotation is that the sample frequencies of the MEMS gyroscope and FOG are different. Thus, a heading error spike will occur if the system has a high rotational speed. The adaptive algorithm estimated the suitable parameters of SF model, so that it could compensate the SF much more accurately than the fixed parameters methods.

Figure 10 and Figure 11 show that the SF has a great influence on the heading precision. The adaptive algorithm has a good performance in simulation, but before it is employed in actual use, there still are some problems that need to be solved, such as the fact the magnetic interference is difficult to identify during rotation, and the errors during rotation caused by SF errors or bias drift are difficult to identify too.

## 5. Conclusions

In this paper, we propose an adaptive algorithm for a system integrated with MEMS gyroscopes and a compass to compensate the bias drift of the MEMS gyroscope accurately in different environments and eliminate the influence of temperature variation. This algorithm only works in an environment without magnetic interference; otherwise, magnetic interference will introduce errors into the system. The proposed algorithm performs much better than KF and MLR, because it uses an STKF to estimate the parameters of the bias model of the MEMS gyroscope with the support of the compass orientation, and it changes these parameters to adapt to the environment intelligently, and then estimates the bias accurately using the model and the parameters. The significant characteristic of this algorithm is that when the algorithm is in an outage for some reason, these appropriate parameters can also maintain high precision to compensate the temperature drift.

This approach can be used easily, and the MEMS gyroscope does not need to calibrate its temperature model before use. As the state variables of the STKF, the parameters of the bias model change with the working environment. Thus, this algorithm has strong adaptability to different work environments and is easy to implement, so it is suitable for middle- or low-level inertial gyroscopes which always work under changing temperature conditions.

## References

[1] Tanaka M. (2007). An industrial and applied review of new MEMS devices features. Microelectron. Eng..

[2] Fang B., Chou W.S., Ding L. (2014). An Optimal Calibration Method for a MEMS Inertial Measurement Unit. Int. J. Adv. Robot. Syst..

[3] Zhang G.Y., Deng Z.L., Fu Z.X. (2003). Temperature modeling study for gyroscope. J. Syst. Simul..

[4] El-Diasty M., El-Rabbany A., Pagiatakis S. (2007). Temperature variation effects on stochastic characteristics for low-cost MEMS-based inertial sensor error. Meas. Sci. Technol..

[5] Kirkko-Jaakkola M., Collin J., Takala J. (2012). Bias prediction for MEMS gyroscopes. IEEE Sens. J..

[6] Zhao L., Wang X.Z., Cheng J.H., Huang C. Research on the High Accuracy Temperature Control Box of FOG. Proceedings of the 29th Chinese Control Conference.

[7] Zhao X., Su Z., Ma X.F., Tian S.X. (2012). Research on bias compensation of MEMS gyroscope under large temperature difference. Chin. J. Sens. Actuators.

[8] Zhao X.H., Yi G.X., Wang C.H. (2008). Research on temperature compensation of silicon gyro’s drift. Transducer Microsyst. Technol..

[9] Cheng L., Wang S.R., Ye F. (2008). Research on bias temperature compensation for micromachined vibratory gyroscope. Chin. J. Sens. Actuators.

[10] Zhou C.L., Zhang Q., Yan S.H., Gao L., Wang G.C. Modeling and Compensation for Temperature errors of Interferometric Fiber Optic Gyroscope. proceedings of the 2009 IEEE International conference on Information and Automation.

[11] Jin J., Wang Z., Zhang Z.G., Song N.F., Zhang C.X. (2008). Temperature Errors Modeling for Fiber Optic Gyroscope Using Multiple Linear Regression Models. J. Astronaut..

[12] Ding J.C., Zhang J., Huang W.Q., Chen S. (2014). Laser Gyro Temperature Compensation Using Modified RBFNN. Sensors.

[13] Song L.M., Zhang W. Temperature Error Compensation for Open-Loop Fiber Optical Gyro using Back-Propagation Neural Networks with Optimal Structure. Proceedings of the 2014 IEEE Chinese Guidance, Navigation and Control Conference.

[14] Zhan R.H., Wan J.W. (2007). Iterated Unscented Kalman Filter for Passive Target Tracking. IEEE Trans. Aerosp. Electron. Syst..

[15] Zha F., Xu J.N., Li J.S., He H.Y. (2013). IUKF neural network modeling for FOG temperature drift. J. Syst. Eng. Electron..

[16] Shen Z., Georgy J., Korenberg M.J., Noureldin A. (2011). Low cost two dimension navigation using an augmented Kalman filter/Fast Orthogonal Search module for the integration of reduced inertial sensor system and Global Positioning System. Transp. Res. Part C.

[17] Ren H.L., Kazanzides P. (2012). Investigation of Attitude Tracking Using an Integrated Inertial and Magnetic Navigation System for Hand-Held Surgical Instruments. IEEE/ASME Trans. Mechatron..

[18] Chen X.Y., Xu Y., Li Q.H. (2013). Application of Adaptive Extended Kalman Smoothing on INS/WSN Integration System for Mobile Robot Indoors. Math. Probl. Eng..

[19] Li W., Wang J.L. (2013). Effective adaptive Kalman filter for MEMS-IMU/magnetometers integrated attitude and heading reference systems. J. Navig..

[20] Borenstein J., Ojeda L. (2010). Heuristic drift elimination for personnel tracking systems. J. Navig..

[21] Fang J.C., Li J.L., Sheng W. (2006). Improved temperature error model of silicon MEMS gyroscope with inside frame driving. J. Beijing Univ. Aeronaut. Astronaut..

[22] Won S.-H.P., Melek W.W., Golnaraghi F. (2010). A Kalman/particle filter-based position and orientation estimation method using a position sensor/inertial measurement unit hybrid system. IEEE Trans. Ind. Electron..

[23] Qin Y.Y., Zhang H.Y., Wang S.H. (1998). Theory of Kalman Filter and Integrated Navigation.

[24] Chen X.Y., Shen C., Zhang W.B., Tomizuka M., Xu Y., Chiu K. (2013). Novel hybrid of strong tracking Kalman filter and wavelet neural network for GPS/INS during GPS outages. Measurement.

[25] Bekkeng J.K. (2009). Calibration of a Novel MEMS Inertial Reference Unit. IEEE Trans. Instrum. Meas..

[26] (1997). IEEE Standard Specification Format Guide and Test Procedure for Single-Axis Interferometric Fiber Optic Gyros.

